# Oral melphalan as a treatment for platinum-resistant ovarian cancer

**DOI:** 10.1038/sj.bjc.6601044

**Published:** 2003-06-10

**Authors:** J Hasan, G C Jayson

**Affiliations:** 1 Cancer Research UK, Department of Medical Oncology, Christie Hospital, Wilmslow Road, Withington, Manchester M20 4BX, UK

**Keywords:** ovary, second line, alkylating agents

## Abstract

A large number of drugs have been used to treat recurrent ovarian cancer, yet there are few data that guide the physician's choice. Typically, the decision to re-treat with platinum-based therapy depends on the progression-free interval. However, the optimum agent for the treatment of platinum-resistant or refractory disease is not defined. In this study, we investigated the efficacy of oral melphalan in patients who have platinum refractory or resistant disease. A retrospective analysis was performed on 22 patients with ovarian carcinomas who had relapsed within 6 months of their platinum-based chemotherapy and were treated with oral melphalan. No objective responses were seen and the median overall survival was 3 months from commencement of therapy. Although the treatment was generally well tolerated, only two of the 22 patients managed to complete the planned six cycles of treatment. At the time of analysis, only two patients were alive. Other nonplatinum compounds have demonstrated response rates in the region of 20% in similar patient populations and it is unlikely that any positive responses could have been missed by chance (95% CI 0–15.4). The results of this study serve to eliminate oral melphalan as a treatment option in patients with platinum-resistant or refractory ovarian carcinoma.

Ovarian cancer is the leading cause of mortality from gynaecological malignancies in the Western world (Greenlee *et al*, 2001). Over 70–80% of patients present with advanced disease (stage III/IV) at diagnosis and the majority of these patients will develop recurrent disease. The most important predictive factor for response at relapse is the progression-free interval (PFI), a surrogate marker for intrinsic chemosensitivity (Blackledge *et al*, 1989; Markman *et al*, 1991). At relapse, patients can be separated into platinum-sensitive (PFI >6 months) and platinum-resistant (PFI <6 months) or platinum-refractory (no PFI) subsets. In general, patients with platinum-sensitive disease can be rechallenged with a platinum compound and nonplatinum agents are reserved for patients with platinum-resistant/refractory disease. Over the last decade, several new compounds have been developed with activity in patients with platinum-resistant/refractory disease. These include the taxanes, topotecan, liposomal doxorubicin and oxaliplatin among others. These agents are associated with response rates in the region of 20% with response durations of 6–8 months in platinum-resistant/ refractory patients (Gore, 2001). In addition, combination treatment regimens have reported higher response rates (Meyer *et al*, 2001; van der Burg *et al*, 2002).

Alkylating agent-based chemotherapy was the treatment of choice for primary epithelial ovarian carcinomas prior to the emergence of the platinum compounds in the 1970s (Brodovsky *et al*, 1984; Klaassen *et al*, 1988; Young *et al*, 1990; Wadler *et al*, 1996). Response rates to oral melphalan ranged from 15 to 40% in advanced disease (stage III/IV). The efficacy of melphalan in the setting of relapsed disease has not been explored sufficiently (Pater *et al*, 1987). In this retrospective analysis, we investigate the effectiveness of oral melphalan as salvage therapy in patients who progressed after platinum-based chemotherapy. The rationale for using melphalan as a second-line agent was its proven efficacy in platinum-naïve patients, ease of administration and tolerability.

## METHODS

The aim of the study was to assess objective tumour response rates in women treated with oral melphalan as second-line therapy after initial platinum therapy for epithelial ovarian cancer and to assess the impact of oral melphalan on relapse-free survival and overall survival in relapsed platinum-resistant epithelial ovarian cancer. All cases of ovarian carcinoma treated with oral melphalan at the Department of Medical Oncology at Christie Hospital between 1995 and 2001 inclusive were retrieved from a computerised database. Oral melphalan was offered to all patients fit to receive treatment. Of the 40 patients treated with oral melphalan, data from 22 patients who had platinum-resistant disease were obtained and retrospectively analysed for the study. Patients who received melphalan as first-line therapy or after two courses of platinum-based chemotherapy (*n*=18) were excluded from analysis. At this time the newer drugs, listed earlier, were not used for the treatment of platinum-resistant disease and therefore most patients who developed early recurrence were treated with melphalan.

Data collected included age at diagnosis, histological subtype of ovarian carcinoma, details of primary treatment including surgery, response rates to primary treatment and PFIs, salvage treatment and treatment-related toxicity. Responses were assessed clinically, radiologically by CT scanning and biochemically by CA125 measurements. Response rates were defined according to WHO criteria.

### Treatment protocol

Melphalan was given orally at 10 mg a day for 5 days repeated every 6 weeks for six cycles. Pretreatment evaluation consisted of a physical examination, full blood count, biochemical profile (renal and liver function tests), CA125 measurements and baseline staging CT. All patients were required to have adequate haematological reserve prior to commencement of treatment. Full blood count and biochemistry were monitored at three weekly intervals. Restaging CT was performed at the end of treatment or where disease progression was suspected on clinical grounds or on the basis of a rising CA125. All patients gave informed consent for the treatment.

Treatment was discontinued after six completed cycles or on account of progressive disease, treatment-related toxicity or patient death. The primary study end point was to determine the percentage of patients who achieve an objective tumour response in terms of a complete response, partial response stable or progressive disease.

## RESULTS

The median age of the 22 patients evaluated was 62 (range 28–78). Four different platinum-based chemotherapeutic regimens were used as first-line therapy – single-agent carboplatin (54.5%), combination chemotherapy consisting of carboplatin/cyclophosphamide (4.5%), carboplatin/paclitaxel (36.3%) or carboplatin/docetaxel (4.5%). The overall response rate to first-line therapy was 68%. The median PFI was 4 months (range 0–6).

The median number of cycles of melphalan administered per patient was 2 (range, 1–6). Only one patient (4.5%) managed to complete the full complement of six cycles and had radiologically proven progressive disease at the end of treatment. No objective radiological responses were observed in any of the 22 patients on the study. As none of the 22 patients experienced an objective response to treatment, no relapse-free survival was observed. In all, 10 patients subsequently went on to third-line chemotherapy or endocrine therapy. The median overall survival from the commencement of oral melphalan was 3 months (range, 0–20 months). A total of 15 (68%) patients died within 6 months of discontinuation of melphalan therapy. At the time of analysis, two our of 22 patients were still alive and these were being treated within the context of phase I/II studies.

In general, oral melphalan appeared to be well tolerated. The 22 patients received 44 completed cycles of oral melphalan. There were seven episodes of grade III/IV haematological toxicity. A total of three episodes of blood transfusions and two episodes of platelet transfusions were recorded. Febrile neutropenic episodes were not seen. Nonhaematological toxicities were infrequent, grade I/II nausea being the most common. No patient was required to discontinue treatment on account of toxicity. No treatment-related mortality was recorded.

## DISCUSSION

In this study, we evaluated the efficacy of oral melphalan as second-line therapy for relapsed epithelial ovarian carcinoma following platinum failure. There were no responses in the study population. Melphalan had no impact on the relapse-free survival or overall survival of patients. However, on closer analysis of the data, some important points emerge. The entire patient population in the study belonged to a poor prognosis group. The most important point is that the median PFI following first-line platinum-based therapy was only 3 months (range 0–6). Thus, all patients had chemoresistant or refractory disease and were therefore likely to fare poorly irrespective of the chemo-therapeutic agent adopted. Given the patient numbers in the platinum-resistant and refractory subgroup, it is unlikely that any objective response would have been missed by chance (95% CI 0–15.44).

Only six patients had undergone optimum debulking surgery following initial diagnosis and the vast majority of patients had a high tumour burden (>2 lesions – 95.4% and >5 cm – 50%), both of which are well-recognised poor prognostic indicators in relapsed disease. However, data from studies with other agents active in platinum-resistant/refractory disease have shown response rates of 20% and response durations of 6–8 months, and although a direct comparison is not possible oral melphalan does appear inferior in this situation. The treatment regimen also needs to be evaluated. The incidence of grade III/IV haematological toxicity in the patient population was 16% with the treatment regimen employed (10 mg daily for 5 days repeated every 6 weeks). Although the treatment in general was well tolerated, a more dose-intense (6–8 mg m^−2^ day^−1^) and a more dose-dense regimen, perhaps a 4-weekly rather than a 6-weekly cycle merits consideration. However, significant myelosuppression was recorded suggesting that this dose and schedule approach the maximum tolerated dose of melphalan.

Despite the retrospective nature of the study, the data suggest that melphalan has poor activity as salvage therapy following platinum failure in relapsed epithelial ovarian cancer and should not be considered for patients with platinum-resistant/refractory disease. Further well-designed randomised controlled trials are needed to define the optimum management for patients with relapsed epithelial ovarian cancers.
